# The Nordic Arthroplasty Register Association: A unique collaboration between 3 national hip arthroplasty registries with 280,201 THRs

**DOI:** 10.3109/17453670903039544

**Published:** 2009-08-01

**Authors:** Leif I Havelin, Anne M Fenstad, Roger Salomonsson, Frank Mehnert, Ove Furnes, Søren Overgaard, Alma B Pedersen, Peter Herberts, Johan Kärrholm, Göran Garellick

**Affiliations:** ^1^The Norwegian Arthroplasty Register, Department of Orthopaedic Surgery, Haukeland University HospitalBergenNorway; ^2^Department of Surgical Sciences, University of BergenNorway; ^3^Locus of Registry Based Epidemiology, Faculty of Medicine, University of BergenNorway; ^4^The Danish Hip Arthroplasty Register, Center for Clinical Databases, Department of Clinical Epidemiology, Aarhus University HospitalAarhusDenmark; ^5^Department of Clinical Epidemiology, Aarhus University HospitalAarhusDenmark; ^6^Department of Orthopaedic Surgery and Clinical Institute, Odense University HospitalOdenseDenmark; ^7^The Swedish Hip Arthroplasty Register. Department of Orthopaedics, Institute of Surgical Sciences, Sahlgrenska University Hospital, University of GothenburgMölndalSweden

## Abstract

**Background and purpose** The possibility of comparing results and of pooling the data has been limited for the Nordic arthroplasty registries, because of different registration systems and questionnaires. We have established a common Nordic database, in order to compare demographics and the results of total hip replacement surgery between countries. In addition, we plan to study results in patient groups in which the numbers are too small to be studied in the individual countries.

**Material and methods** Primary total hip replacements (THRs) from 1995–2006 were selected for the study. Denmark, Sweden, and Norway contributed data. A common code set was made and Cox multiple regression, with adjustment for age, sex, and diagnosis was used to calculate prosthesis survival with any revision as endpoint.

**Results** 280,201 operations were included (69,242 from Denmark, 140,821 from Sweden, and 70,138 from Norway). Females accounted for 60% of the patients in Denmark and Sweden, and 70% in Norway. Childhood disease was the cause of 3.1%, 1.8%, and 8.7% of the operations in Denmark, Sweden, and Norway, respectively. Resurfacing of hips accounted for 0.5% or less in all countries. The posterior approach was used in 91% of cases in Denmark, 60% in Sweden, and 24% in Norway. Cemented THRs were used in 46% of patients in Denmark, in 89% of patients in Sweden, and in 79% of patients in Norway.

Of the 280,201 primary THRs, 9,596 (3.4%) had been revised. 10-year survival was 92% (95% CI: 91.6–92.4) in Denmark, 94% (95% CI: 93.6–94.1) in Sweden, and 93% (95% CI: 92.3–93.0) in Norway. In Denmark, 34% of the revisions were due to dislocation, as compared to 23% in Sweden and Norway. Replacement of only cup or liner constituted 44% of the revisions in Denmark, 29% in Sweden, and 33% in Norway.

**Interpretation** This unique common Nordic collaboration has shown differences between the countries concerning demographics, prosthesis fixation, and survival. The large number of patients in this database significantly widens our horizons for future research.

## Introduction

The Nordic countries, including Denmark, Sweden, Finland, and Norway, have all had a long and successful tradition of arthroplasty registries (Herberts et al. 1989, Lucht 2000, Havelin et al. 2000, Pedersen et al. 2004, Malchau et al. 2005, Puolakka et al. 2001).

The annual reports from the Nordic registries have indicated differences between the countries concerning choices of implant brands, fixation methods, and implant survival. However, because of differences in registration parameters, statistical methods, and patient selection, the results found in the annual reports from each country have not been fully comparable. Furthermore, the impact of the Nordic registries on improvements in total hip replacement (THR) surgery, which from an international perspective are based on relatively small patient populations, is inherently limited by specific demographics, implant selection, and treatment traditions. Thus, there is a need for collaboration across national borders to enable extended analyses and give more reliable outcome data.

Knowing the limitations of registries with small populations, representatives from the artroplasty registries in Denmark, Finland, Norway, and Sweden met and decided to collaborate in order to establish a common database covering selected data regarding THR procedures. During the process, the Finnish registry decided not to participate in the present study, due to changes in its staff. The aim of this particular study was to compare demographics, choice of implant, fixation techniques, and results between the 3 remaining countries.

## Materials and methods

### Sources of data

The hip arthroplasty registries of Sweden, Denmark, and Norway participated in the present study. The Swedish Hip Registry was established in 1979, whereas the Norwegian Arthroplasty Registry and the Danish Hip Registry started registration in 1987 and 1995, respectively. From 1995, all 3 registries have used individual-based registration of operations and patients. We therefore decided to select primary THRs performed during 1995–2006 for the present study.

The databases in the 3 registers were not fully compatible, as we had different registration forms including somewhat different variables, and to some extent also different definitions of variables. Thus, we defined a common set of parameters, containing only data that all 3 registries could deliver and consensus was made according to definition of several variables. However, for cement and prosthesis brands we kept the national codes unchanged but coupled them to additional country codes.

Selection and transformation of the respective data sets and de-identification of the patients, including deletion of the national civil registration numbers, were performed within each national registry. Anonymous data were then merged into a common database.

Data were treated with full confidentiality, according to the rules of the respective countries. This included access to the common database, which was limited to the co-authors of the present paper. It is not possible to identify patients at an individual level, either in this paper or in the database.

### Statistics

Descriptive statistics and age- and sex-specific incidence rates for the 3 countries were calculated. Differences in patient and procedure characteristics between the 3 countries were tested using the chi-square test. A table with the 10 most commonly used combinations of cup and stem was constructed for each country, classifying the components after brand name regardless of other properties such as material and design. Survival curves were constructed by the Kaplan-Meier method, and all curves were ended when the number of patients at risk was below 100. Patients were censored at death or outcome, whichever came first. Outcome was any revision, defined as removal or exchange of at least one of the components. Only revisions with the primary THA recorded in the registries were included as outcome. We used Cox multiple regression models to assess survival and relative risk (RR) of any revision as endpoint, with 95% confidence interval (CI) and with adjustment for age, sex, and diagnosis.

For hybrids and uncemented THRs, we performed additional time-dependent survival analyses because the country curves crossed each other indicating that the conditions for Cox regression were not fulfilled during the total time period. In these analyses on uncemented THRs, the follow-up after THR was divided into 2 periods. The first period was defined from the day of surgery until 5 years after surgery and the second period commenced at 5 years after surgery and ran until December 31, 2006. For hybrids, the follow-up period was divided at 7 years after surgery.

The statistics packages SPSS version 15.0 (SPSS Inc., Chigaco, IL) and S-Plus 7.0 (Insightful Corp., Seattle, WA) were used for the analyses.

The study was approved by The Danish Data Protection Agency, J. no. 2008-41-2024, and by the Norwegian Social Science Data Services.

## Results

280,201 operations fulfilled the inclusion criteria for the study (69,242 in Denmark, 140,821 in Sweden, and 70,138 in Norway).

### Demographics

Females constituted 58% of the patients in Denmark, 60% in Sweden, and 70% in Norway. For male patients over the age of 50, the incidence rate of THR was lower in Norway than in the other countries whereas the Norwegian female patients had the highest incidence rate of THR in all age groups (Table 1).

**Table 1.** Age and sex-specific incidence rates from 1995–2006, of primary total hip arthroplasties per 100,000 inhabitants

	Denmark		Sweden		Norway	
Age	Male	Female	Male	Female	Male	Female
10–49	14	11	11	12	11	15
50–59	120	116	127	143	103	175
60–69	323	366	340	414	278	557
70–79	466	611	516	696	452	928
≥ 80	369	435	373	481	347	560

### Hip disease

Childhood diseases accounted for a larger proportion of the patients in Norway (8.6%) than in Denmark and Sweden (3.1% and 1.8%), and the proportion of patients operated due to idiopathic necrosis of the femoral head was higher in Denmark (2.8%) and Sweden (2.9%) than in Norway (1.3%). Furthermore, the proportion of patients who were operated due to inflammatory arthritis was highest in Sweden (3.6%), slightly lower in Norway (3.3%), and lowest in Denmark (2.4%) (Table 2).

**Table 2.** Characteristics of the total hip arthroplasty patients and operations registered in the NARA database, 1995–2006

	Denmark	Sweden	Norway	p-value ^a^
No. of THAs	69,242	140,821	70,138	
Male sex, %	41.7	39.8	30.2	< 0.001
Age groups, %				
< 30 years	0.5	0.3	0.5	
30–39	1.4	1.0	1.3	
40–49	4.2	3.5	3.8	
50–59	14.7	13.7	12.7	
60–69	29.9	28.0	26.4	
70–79	34.1	36.5	39.1	
≥ 80 years	15.2	17.1	16.3	< 0.001
Diagnosis, %				
Primary osteoarthritis	77.6	78.8	73.7	
Inflammatory arthritis	2.4	3.6	3.3	
Hip fracture	11.9	11.5	11.2	
Childhood diseases	3.1	1.8	8.6	
Idiopatic caput necrosis	2.8	2.9	1.3	
Others	2.3	1.3	2.0	< 0.001
Fixation, %				
Cemented	45.9	88.9	78.9	
Uncemented	26.8	4.4	13.2	
Hybrid	26.4	4.0	4.0	
Inverse hybrid	0.6	2.2	3.8	
Resurfacing	0.3	0.5	0.2	< 0.001
Approach, %				
Posterior	91.0	60.3	23.8	< 0.001
Cause of revision, %				
Aseptic loosening	34.8	50.4	47.3	
Deep infection	15.8	15.0	15.5	
Perioprostetic fracture	5.1	6.7	3.5	
Dislocation	33.5	23.4	23.8	
Pain only	3.0	0.8	2.3	
Others	7.8	3.7	7.6	< 0.001
Procedure at revision, %				
Total prosthesis replaced	16.4	28.4	21.6	
Only stem replaced	20.7	24.8	27.5	
Only cup replaced	43.7	29.6	32.9	
Extraction of the total prosthesis	8.9	8.6	9.1	
Others	10.3	8.6	8.9	< 0.001

^a^ Chi-square test for differences between countries.

### Prosthetic fixation technique and surgical approach

The largest differences in surgical policies were in relation to fixation technique and surgical approach (Table 2). In Sweden and Norway, most THRs were cemented; that is, cemented THRs were used in 89% and 79% of the patients in these countries whereas they were used in only 46% of the patients in Denmark.

We found that in Denmark the use of uncemented implants had been increasing during the study period, and the use of hybrids (cemented stem and uncemented cup) had been decreasing, whereas in Sweden and Norway the use of inverse hybrids (uncemented stem and cemented cup) had increased.

A posterior approach was used in 91% of the cases in Denmark, 60% in Sweden, and 24% in Norway. A lateral surgical approach, without osteotomy of the major trochanter, was the most common approach in Norway. A lateral approach with a trochanteric osteotomy was more common in Norway (3.7%) than in the other countries (0.9% in Denmark and 0.2% in Sweden). A minimally invasive incision (MIS) was uncommon in all countries (2.2% in Denmark, 0.1% in Norway, and 0.2% in Sweden).

### Brands of prosthesis

We found substantial differences in choices of prosthesis brands between the 3 countries (Table 3). Only the Charnley/Charnley (DePuy) cemented, Exeter/Exeter (Stryker) cemented, and Lubinus or IP (Link)/SP II (Link) cemented cup/stem combinations were on the top-ten list in all 3 countries, and of the rest, only the combination of Reflection cemented cup/ Spectron EF stem (Smith and Nephew) was among the top 10 in more than one of the countries. Resurfacing constituted 0.5% or less of the procedures in all 3 countries.

**Table 3.** The 10 most commonly used total hip prostheses (combination of cup/stem) in each country, according to brand and type of fixation

Cup	Stem	Fixation	n
*Denmark*			
Lubinus/IP (Link) **^a^**	SP II (Link)	Cemented	7,652
Exeter (Stryker)	Exeter (Stryker)	Cemented	6,779
Trilogy (Zimmer)	Bimetric (Biomet)	Uncemented	6,299
Trilogy (Zimmer)	Bimetric cemented (Biomet)	Hybrid	4,422
ZCA (Zimmer)	CPT (Zimmer)	Cemented	3,233
Universal (Biomet)	Bimetric cemented (Biomet)	Hybrid	2,912
Müller (Biomet)	Bimetric cemented (Biomet)	Cemented	2,548
Charnley (DePuy)	Charnley (DePuy)	Cemented	2,533
Mallory-Head (Biomet)	Bimetric (Biomet)	Uncemented	2,400
Mallory-Head (Biomet)	Exeter (Stryker)	Hybrid	1,628
*Sweden*			
Lubinus/IP (Link) **^a^**	SP II (Link)	Cemented	48,659
Exeter (Stryker)	Exeter (Stryker)	Cemented	15,383
Charnley (DePuy)	Charnley (DePuy)	Cemented	14,590
Reflection Cemented (S & N)	Spectron EF (S & N)	Cemented	6,946
Charnley Elite (DePuy)	Exeter (Stryker)	Cemented	6,549
FAL (LINK)	SP II (Link)	Cemented	4,059
Contemporary (Stryker)	Exeter (Stryker)	Cemented	2,545
Charnley (DePuy)	Exeter (Stryker)	Cemented	2,095
OPTICUP	Scan Hip II	Cemented	1,981
Charnley (DePuy)	Elite (DePuy)	Cemented	1,405
*Norway*			
Charnley (DePuy)	Charnley (DePuy)	Cemented	22,591
Reflection Cemented (S & N)	Spectron EF (S & N)	Cemented	6,906
Exeter (Stryker)	Exeter (Stryker)	Cemented	6,765
Titan (DePuy)	Titan (DePuy)	Cemented	3,943
Lubinus/IP (Link) **^a^**	SP II (Link)	Cemented	1,394
Tropic (DePuy)	Corail (DePuy)	Uncemented	1,711
Igloo (Biotechni)	Filler (Biotechn)i	Uncemented	1,554
Kronos (DePuy)	Titan (DePuy)	Cemented	1,201
Contemporary (Stryker)	Exeter (Stryker)	Cemented	960
Elite (DePuy)	Titan (DePuy)	Cemented	922

^a^ Due to the coding systems, the cups IP (Link) and Lubinus (Link) were pooled.

S&N = Smith & Nephew

### Survival and revisions (Figure 1)

Of the 280,201 primary THRs inserted during the study period, 9,596 (3.4%) had later been revised (Table 4). The overall 10-year survival of all THRs was 92.0% (95% CI: 91.6–92.4) in Denmark, 93.9% (95% CI: 93.6–94.1) in Sweden, and 92.7% (95% CI: 92.3–93.0) in Norway, with any revision as endpoint. The adjusted overall RR for any revision was 0.66 (95% CI: 0.63–0.69) and 0.84 (95% CI: 0.79–0.88) in Sweden and Norway compared to Denmark (Table 4).

**Figure 1.**
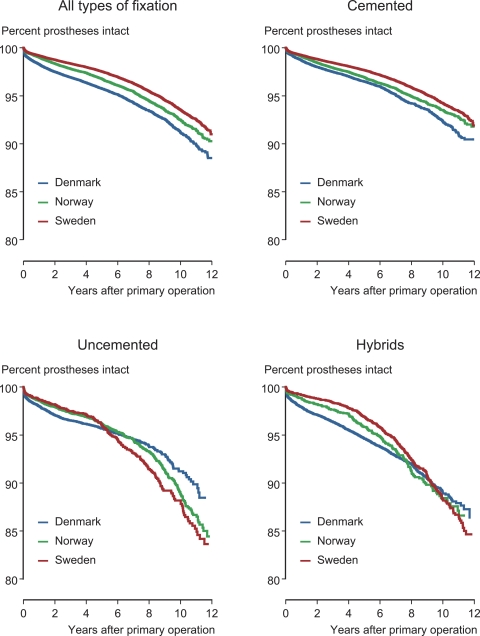
Kaplan-Meier estimated curves until revision for any cause, for primary total hip replacements (THAs) in Denmark, Sweden, and Norway 1995–2006. Curves are given for all THAs and also for those classified according to fixation technique as cemented, uncemented, and hybrids (uncemented cup/cemented stem).

**Table 4.** Cox regression estimates of survival probabilities and relative risk of any revision (RR) with 95% confidence intervals (CIs) for all THAs and according to fixation technique with adjustment for sex, age, and diagnosis, and comparing the three countries. Subanalyses on patients younger and older than 60 years of age are presented

	No. of			Cox		Survival (%) at 10 years	K-M 95% CI	
	Country	THRs	Revisions	RR	95% CI	p-value
*All age groups*								
All THAs	Denmark	69,195	3,006	1			92.0	91.6–92.4
	Sweden	140,589	4,001	0.66	0.63–0.69	< 0.001	93.9	93.6–94.1
	Norway	69,732	2,554	0.84	0.79–0.88	< 0.001	92.7	92.3–93.0
Cemented THAs	Denmark	31,743	1,298	1			92.9	92.4–93.4
	Sweden	124,701	3,277	0.68	0.64–0.72	<0.001	94.7	94.5–94.9
	Norway	54,709	1,823	0.90	0.84–0.97	0.005	93.5	93.2–93.9
Uncemented THAs	Denmark	18,518	683	1			91.5	90.4–92.6
	Sweden	6,133	263	1.04	0.90–1.20	0.6	88.2	86.4–90.0
	Norway	9,142	483	0.94	0.83–1.07	0.4	89.4	88.2–90.6
Hybrids	Denmark	18,299	1000	1			89.1	88.2–90.1
	Sweden	5,602	348	0.74	0.64–0.84	<0.001	89.8	88.5–91.1
	Norway	2,745	183	0.92	0.78–1.08	0.3	88.4	86.4–90.3
*Patients < 60 years of age*								
All THAs	Denmark	14,367	829	1			89.1	88.2–90.1
	Sweden	25,917	1203	0.82	0.75–0.90	<0.001	89.4	88.7–90.1
	Norway	12,745	668	0.90	0.81–1.00	0.05	88.2	87.1–89.3
Cemented THAs	Denmark	2,303	196	1			87.5	85.6–89.4
	Sweden	15,272	643	0.68	0.57–0.79	<0.001	91.2	90.4–92.1
	Norway	4,994	241	0.89	0.73–1.08	0.2	89.1	87.3–90.8
Uncemented THAs	Denmark	8,645	360	1			91.0	89.6–92.4
	Sweden	4,646	228	1.12	0.94–1.33	0.2	87.6	85.7–89.5
	Norway	5,388	334	0.97	0.83–1.14	0.7	88.5	87.0–90.0
*Patients* ≥ *60 years of age*								
All THAs	Denmark	54,828	2,177	1			92.5	92.1–92.9
	Sweden	114,672	2,798	0.59	0.56–0.63	<0.001	94.9	94.7–95.2
	Norway	56,987	1,886	0.81	0.77–0.87	<0.001	93.6	93.2–94.0
Cemented THAs	Denmark	29,440	1,102	1			93.5	93.0–94.0
	Sweden	109,429	2,634	0.68	0.63–0.73	<0.001	95.1	94.9–95.4
	Norway	49,715	1,582	0.92	0.85–0.99	0.03	93.9	93.6–94.3
Uncemented THAs	Denmark	9,873	323	1			92.9	91.3–94.5
	Sweden	1,487	35	0.74	0.52–1.06	0.1	92.3	88.5–96.1
	Norway	3,754	149	0.94	0.77–1.16	0.6	92.5	90.8–94.2

Sweden had the highest survival rates of cemented and hybrid prostheses. In patients younger than 60 years of age, we found better overall survival of THRs in Sweden than in Denmark (adjusted RR was 0.82 (95% CI: 0.75–0.90)), but it was not better than in Norway (adjusted RR of 0.90 (95% CI: 0.81–1.0)) (Table 4). For patients older than 60 years of age, the overall survival of THRs was better in both Sweden and Norway than in Denmark, with adjusted RRs of 0.59 (95% CI: 0.56–0.63) and 0.81 (95% CI: 0.77–0.87) (Table 4).

**Figure 2.**
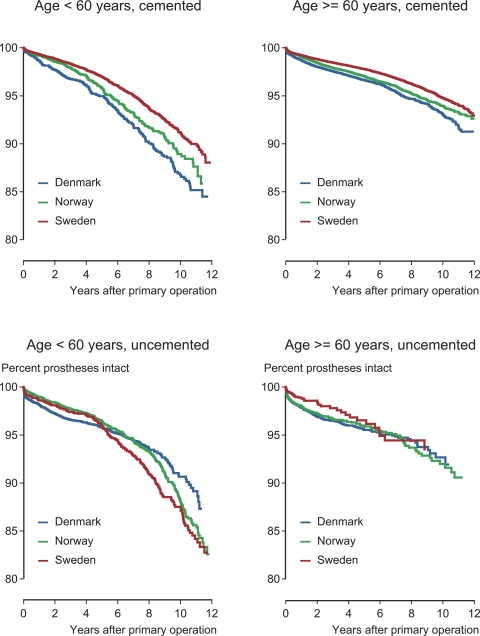
Kaplan-Meier estimated curves until revision for any cause, for primary cemented and uncemented total hip replacements (THRs) in Denmark, Sweden, and Norway 1995–2006, in patients younger than 60 years and in those aged 60 and older.

We found a 20% reduced risk of revision for uncemented THRs in Sweden and Norway during the first 5 years of follow–up, compared with Denmark (Table 5). However, if patients did not undergo any revision during the first 5 years after surgery, the survival of uncemented THRs was better in Denmark than in Sweden and Norway (adjusted RR was 2.15 (95% CI: 1.63–2.83) for Sweden and 1.61 (95% CI: 1.26–2.06) for Norway) (Table 5). The same pattern was observed for uncemented implants in patients less than 60 years of age (Table 5 and Figure 2).

**Table 5.** Cox regression estimates of survival, and relative risk of any revision (RR) with 95% confidence intervals (CIs), according to time-dependent covariates (follow-up < 5 years or ≥ 5 years for uncemented THAs, and follow-up < 7 years or ≥ 7 years for hybrids), for all age groups and for patients less than 60 years of age

	RR	95% CI	p-value	RR	95% CI	p-value
*Uncemented THA, all age groups:*	*Follow-up < 5 years*			*Follow-up* ≥ *5 years*		
Denmark	1			1		
Sweden	0.79	0.66–0.95	0.013	2.15	1.63–2.83	< 0.001
Norway	0.80	0.69–0.93	0.003	1.61	1.26–2.06	< 0.001
*Hybrids, all age groups:*	*Follow-up < 7 years*			*Follow-up* ≥ *7 years*		
Denmark	1			1		
Sweden	0.59	0.50–0.68	< 0.001	1.48	1.15–1.92	0.003
Norway	0.83	0.69–1.00	0.051	1.47	1.05–2.06	0.03
*Uncemented THA, age < 60 years:*	*Follow-up < 5 years*			*Follow-up* ≥ *5 years*		
Denmark	1			1		
Sweden	0.86	0.62–0.93	0.007	1.96	1.45–2.64	< 0.001
Norway	0.76	0.69–0.93	0.2	1.60	1.22–2.10	0.001

We also found better survival of hybrid THRs in Sweden and Norway than in Denmark within 7 years of surgery, whereas if patients did not undergo revision within 7 years, the survival of hybrid implants was again better in Denmark.

In Denmark, 34% of revisions were due to dislocation, as compared to 23% in Sweden and Norway (Table 2). Replacement of only the cup or liner accounted for 44% of the revisions in Denmark, 29% in Sweden, and 33% in Norway (Table 2). The percentage of extractions of the total prosthesis, temporarily or permanently—which is a common treatment for septic hip prostheses—was virtually the same in the three countries.

## Discussion

To the best of our knowledge, this is the first time data from 3 national arthroplasty registries have been successfully merged. The main findings in the present paper were the large differences between the three countries concerning prosthesis brands, fixation methods, and surgical approach. Thus, uncemented implants and the posterior approach were used more commonly in Denmark than in the other countries. We also found that more female patients and fewer male patients were operated in Norway than in the other countries, and that the proportion of patients operated due to certain hip diseases differed between the countries. Concerning prosthesis survival, the differences were relatively minor, but we found different patterns in the 3 countries concerning reasons for revision and procedures performed at revision.

### Demographics

We found different incidences of THR in the 3 countries. This may be due to different availability of THR, or may be for genetic reasons (Ingvarsson 2000). The finding of more female patients in Norway than in the other 2 countries is in accordance with the work of Lohmander et al. (2006). Furthermore, we found that a greater proportion of the Norwegian patients were operated with THR due to childhood hip diseases than in the other 2 countries. There could be a relationship between these 2 findings, as dysplasia is more common in girls than boys. Childhood hip diseases may be more common in Norway, or alternatively, the diagnostics and treatment of these diseases in the now aging population may have been inferior in Norway compared to the other countries when these patients were children.

The percentage of patients with avascular necrosis of the femoral head was low in all 3 countries compared to the rest of Europe and the USA, but the incidence was more than twice as high in Denmark and Sweden as in Norway (Mont and Hungerford 1995). There may be genetic reasons for this (Zalavras et al. 2004). The ethnic ties between continental Europe and Denmark and Sweden may have been closer than for Norway. In addition, several other factors have been recognized as risk factors for avascular head necrosis, including steroid therapy, alcohol consumption, trauma in childhood, and chemotherapy. The impact of these factors on development of avascular necrosis may differ between the Nordic countries. Furthermore, alcohol drinking habits in Denmark and Sweden may be more similar to those of continental Europe and the USA.

Finally, validation of hip diagnosis for primary THRs has been done in Denmark through review of medical records, but not in the 2 other countries (Pedersen et al. 2004). Thus, the differences in diagnosis distribution, including the high proportion of childhood diseases in Norway, may also be related to different definitions of diagnosis and reporting in the different countries.

### Prosthesis brands and fixation

The differences in choices of prosthesis brands in the 3 countries are difficult to explain. To a certain extent, they are probably effects of scientific evidence collected over the years, influenced to a greater or lesser degree by tradition and marketing policies governed by the manufacturers.

The high percentage of cemented prostheses in Sweden and Norway may be explained by the earlier establishment of hip registers in these countries, compared to Denmark. There have been few reasons for Swedish orthopedic surgeons to change from cemented to uncemented prostheses, as the Swedish registry has shown good results with cemented prostheses and has shown that Sweden has had satisfactory results compared to all other countries where data is available (Herberts and Malchau 1997, 2000).

In Norway, since the mid 1990s the registry has had several publications showing inferior results with uncemented prostheses, especially using the uncemented cups with UHMWPE liners, compared to cemented all-polyethylene cups (Havelin et al. 1994, 1995, 2000, 2002). Based on these publications, the Norwegian orthopedic surgeons stopped using the uncemented implants with documented inferior results, but many of these surgeons converted to another uncemented implant instead of changing over to cemented implants.

In Denmark, the orthopedic surgeons seemingly follow the same trends as in most continental western European countries and in the USA, with an increased use of uncemented prostheses during the last third of the study period. As the present study did not include articulation materials as a parameter, we cannot tell whether these uncemented prostheses included cups with UHMWPE liners, or if articulations based on highly crosslinked polyethylene, metal-on-metal, or ceramic-on-ceramic were used. However, for modular uncemented cups with UHMWPE liners and a stiff metal back, there have been very few, if any, publications showing good medium-term or long-term results. For some uncemented cup brands with UHMWPE, but with other types of metal backing than those predominating in the present material, the results seem to be better (Reigstad et al. 2008).

### Prosthetic survival

The absolute differences in survival of prostheses between the 3 countries were minor. However, with such large numbers of patients as in the present study, even a 1% difference in survival at 10 years might be statistically significant.

We found that Sweden and Norway, with their high proportion of cemented prostheses, had better overall survival results than Denmark, which had the highest proportion of uncemented implants. Time-dependent analyses revealed that these findings applied especially to uncemented and hybrid implants within 5 and 7 years of surgery, whereas the results were opposite in the second follow-up period from 5 or 7 years after THR surgery and beyond. This finding may be partly related to the higher frequency of revision in Denmark due to dislocations, which commonly occur within a year after surgery. Even so, the finding may be related to the choices of uncemented prosthesis brands in the 3 countries, resulting in different failure patterns.

### Revisions

The higher percentage of revisions due to dislocations in Denmark might possibly reflect the more common use of a posterior approach in Denmark, compared to Sweden and Norway. In most publications, a higher risk of dislocation has been found with the posterior surgical approach than with alternative approaches (Arthursson et al. 2005). Other factors such as the choice of implant brand, offset, head diameter, and other aspects of the surgical technique, might also have had an effect on the dislocation rate (Byström et al. 2003, Berry et al. 2005). However, head diameter was not a parameter in the Nordic database in the present study. The higher percentage of exchange of cup or liner in Denmark than in Sweden and Norway may reflect the more extensive use of uncemented cups in Denmark. Liner exchange has been a common reason for revision in the case of uncemented cups with UHMWPE liners, and for some hydroxyapatite-coated uncemented cups there has been a high rate of revision due to aseptic loosening (Havelin et al. 2002). In the present study, however, we could neither differentiate liner exchange from cup exchange as the revision procedure, nor differentiate cup loosening from cup wear as the reason for revision. In future studies, we will address these endpoints and issues more thoroughly.

### Study model and future studies

The basis of the database used in the present study was the 3 national databases in the respective countries that keep records of different parameters. When merging data into a common database, we could only include parameters that all 3 registries could contribute. Thus, the Nordic database is not as rich in detail as each separate database. Furthermore, for similar reasons, we could not include THRs from before 1995, which imposed limitations in the length of follow-up-time. On the other hand, the strength of this Nordic project is that we, for the first time, can directly compare the descriptive statistics and the survival results of THRs between three countries. The Nordic project may be looked upon as a start on the long way to assisting in the development of standardized registry data and approaches to analysis, which could help in developing cooperation between registries internationally in the future.

We used survival analysis with adjustment for demographic differences rather than revision burden, as we believe that the use of survival analysis is a more accurate way of presenting and comparing the results of THR surgery. As described in an annual report from the Danish Hip Arthroplasty Registry (Annual Report 2007, www.dhr.dk), the term revision burden can be defined in at least three different ways. For the time being, there is no consensus on definition of revision burden between European national registries. By using survival analysis, we are able to avoid definition issues. In addition, the rapidly increasing amount of prosthesis surgery in some countries, with accordingly small numbers of prostheses with long follow-up in the population, would have a large effect on revision burdens. Revision burdens might thus be frequently inaccurate and misleading, and they should be used with caution and only be presented together with thorough explanations of their flaws. Nevertheless, in comparing national results by means of survival analysis or revision burdens, the data should be collected, selected, and validated similarly in the different countries—and these data should preferably be pooled before national results are assessed and compared.

For all the national registries that delivered data to the common Nordic database, there is documentation on the high degree of completeness, which reduces the selection bias and increases the generalizability of the results (Sødermann 2000, Pedersen et al. 2004, Arthursson et al. 2005, Espehaug et al. 2006).

As there appears to be a tendency for most orthopedic surgeons to follow the most accepted surgical policies in their respective countries, there have been limitations within each country on comparing the mainstream treatment with the alternative treatments that might have been used in smaller numbers of patients. As mentioned above, however, concerning surgical approach and the use of uncemented implants, the mainstream treatment differs between the 3 countries. Thus, regarding future studies, we look forward to comparing results with satisfactory statistical strength, between groups of patients that would be too small to study in each country alone. It would also be interesting to compare the different national results with a particular commonly used prosthesis brand, such as the Charnley, Exeter, Lubinus/SP II, or Reflection cemented/Spectron. Such studies could probably provide an indication of the quality of surgery per se, and of the quality of education programs for orthopedic surgeons in training.

With this Nordic database, we also plan to follow the results of new treatments such as resurfacing prostheses and articulations without UHMWPE, and the results of THRs in smaller age groups or diagnostic groups.

Prostheses of the knee and patients treated with prostheses due to proximal femoral fractures will also be subjects for future studies. In these studies, we expect that Finland will also join in.

### Conclusion

This unique collaboration of the Nordic national orthopedic databases is now functional, with three countries participating so far, and it has shown that there are differences between the countries concerning demographics, prosthesis fixation, prosthesis survival, and revision patterns. The large numbers of patients in this database widens our horizons for future research, and we expect that this new register will become a valuable tool in our future research with the aim of improving the quality of joint replacement surgery.
